# Nanocarbon-Iridium Oxide Nanostructured Hybrids as Large Charge Capacity Electrostimulation Electrodes for Neural Repair

**DOI:** 10.3390/molecules26144236

**Published:** 2021-07-12

**Authors:** Nieves Casañ-Pastor

**Affiliations:** Solid State Chemistry Department, Institut de Ciencia de Materials de Barcelona, CSIC, Campus UAB, 08193 Bellaterra, Spain; nieves@icmab.es; Tel.: +34-935-801-853

**Keywords:** nanocarbons, iridium oxide, charge capacity, electrostimulation, repair

## Abstract

Nanostructuring nanocarbons with IrO_x_ yields to material coatings with large charge capacities for neural electrostimulation, and large reproducibility in time, that carbons do not exhibit. This work shows the contributions of carbon and the different nanostructures present, as well as the impact of functionalizing graphene with oxygen and nitrogen, and the effects of including conducting polymers within the hybrid materials. Different mammalian neural growth models differentiate the roles of the substrate material in absence and in presence of applied electric fields and address optimal electrodes for the future clinical applications.

## 1. Introduction

Carbon plays a unique role in electrochemical catalysis and energy storage systems or sensors. Its contribution to conductivity and its intrinsic capacitance has been explored in capacitors and batteries, alone and in combination with other components [1]. In particular, the additional faradaic contributions from other components in nanostructured hybrid materials have been crucial to develop new nanostructures and also to improve supercapacitor electrodes as nicely reviewed in [2,3,4]. Furthermore, when in form of graphene or nanotubes (CNT), nanocarbons show added electrochemical features with respect to graphitic carbons [5].

From a biological point of view, carbons have been shown to be good substrates for cell growth [6,7], and in the case of nanocarbons like carbon nanotubes or single-layer graphene coatings, also allow sensing, drug delivery, imaging and local electrostimulation [8,9]. However, despite all expectations derived from graphene high conductivity, no evidence of charge capacity in isolated nanocarbons has been given. Electrostimulation has neither been possible to date in those cases, despite the reported interactions. Carbon nanotubes on the other hand have mixed reports of biocompatibility. It is worth noting that CNT are known to enter cells, evidencing phagocytosis, which allows labelling in medical applications, and are toxic in some reports [10]. On the other hand, any type of nanoparticle ends up within cells, with a variety of responses. While this is useful for detection of cancer cells, the time viability of healthy cells is not yet known.

In some cases, simplified interpretations of the role of nanocarbons have been given, like the possible explanation that graphene surfaces modify the mobility of K^+^ ions and in turn enhance neuron excitability [11] While possible, no proof exists of that, since potassium is present mostly internally in cells and the extra cellular space has higher concentrations of other ions. In fact, previously, it has been shown that it is Ca^2+^ concentration the main factor modifying the direction of neural growth [12].

Furthermore, the conducting character of carbon and nanocarbon particles is significant beyond the usual reports. Induced electrical dipoles in conducting materials are basically ignored in most works related with neural development. However, recent works have shown [13,14,15] that conducting materials immersed in cell cultures undergo dipole formation in presence of external fields. Such dipoles, in turn, induce also additional effects in cell behavior and cell growth, and open the possibility of remote electrostimulation protocols. Despite the similar physical wireless effects, each type of material has a different effect on neurons, even with similar conductivity and redox behavior. Thus, PEDOT-PSS (Poly(3,4-ethilendioxithiophene)-poly(estyrene sulfonate)) conducting polymers modify neurite growth direction, while IrO_x_ enhances the speed of dendrite growth. [13]. Although the possible wireless effect is present for any conducting material, refs [13,14,15] it is not yet known why different materials differ in the type of effects on neural cells.

On the other hand, what experiments suggest [16] is that the properties of nanoparticles, including carbon-graphene, may be different in bulk as part of nanostructured materials than in isolated form. Nanoparticles in suspension migrate to the interior of cells, and for that reason are interesting labelling agents and therapeutics for cancer, but such migration would be hindered if nanoparticles are part of a hybrid material. Therefore, beyond the intrinsic effects for nanocarbons, or the effect related with their size, conforming them into nanostructured materials offers a new route to interact with biological systems. To start, bulk nanostructured materials may allow the formation of micro and macro electrodes with specific exposed surface areas, and porosity that allows cell oxygenation and vascularization.

Surface exchange effects may be modified by enhancing surface area, and so three-dimensional scaffolds have been created based on carbon-based structures such as graphene oxide or reduced graphene oxide [17]. These 3D structures allow growth and vascularization through them, and may reach eventually a good electrical connection that allows stimulation. Carbon fibers have also been used in solid arrays with conducting polymers coating them, although the final electrochemistry seems to be only that of the polymer. Although conducting polymers may seem an alternative, either as support of carbon or as coating, neither the biocompatibility nor the electrochemical behavior is improved by forming the composite [18].

Electrostimulation experiments have not been reported yet in either of those nanocarbon cases as far as we know, neither on 3D carbon scaffolds or carbon fibers, although sensing has been possible with single nanotubes [7]. Usual electrostimulation has been carried out clinically with bare metals (steel, platinum, and TiN) [19], coated in some cases with Iridium oxide with a fast electrical response not found in carbons.

Electrodeposited IrO_x_ is, on the other hand the best substrate for neural growth [19,20] and with good conductivity. When used as coating for those basic electrodes an enhancement of charge capacity and decreasing inflammation in the biological tissue is observed. Recent studies have shown that IrO_x_ substrates favor the optimal adhesion of neurons and dendrite growth. It is suspected that redox intercalation of ions in the presence of electric fields, and ionic compositional gradients modify cell behavior [21].

Conducting polymers have also been studied, with polypyrrole-X and PEDOT-X conducting polymers using various counterions, X, [22], being X a biocompatible counterion. The idea is based on the fact that the conductivity of such polymers may offer an alternative to the conductivity of carbon materials, while allowing easy conformation as fibers or 3D substrates. However, the work has not offered yet the expected results. If X is the usual commercial PSS (polysterene sulfonate), contradictory results are found, depending on the adhesion layer used for cell adhesion (polylysine, collagen, etc.) [18]. Additionally, different cell types behave differently on them. While primary mammalian neurons do not grow on PEDOT-PSS, astrocytes do [18], and also xenopus neurons [13]. Biocompatibility is clear for mammalian neurons; however, if aminoacids such as lysine are used as X counterions [22], the role of X in the base polymer is evidenced.

Once materials with optimal compatibility are obtained, a crucial need for all those electrodes is a significant value of charge capacity, that allows a safe charge delivery while maintaining optimal biocompatibility in absence and in presence of electric fields. That may be achieved by the creation of a large surface area through formation of 3D structures as mentioned above that will offer an enhanced charge transfer at the surface, or through generation of nanostructures with several biocompatible components, that, in combination, show better properties than the sum from each component. In both approaches, an enhanced surface interacting with the biosystems and larger charge capacity are expected to decrease inflammation and electric field secondary effects.

As mentioned, graphene oxide and reduced graphene oxide have been prepared in macroscopic solid 3D forms using directional freeze drying processes, and evidencing significant compatibility effects and reinforcing neural cell growth and metabolism. However, in electrochemical terms, no significant conductivity or charge capacity has been achieved yet. On the other hand, the intrinsic properties of the starting graphene materials used are a key factor, and pristine graphene has properties above graphene oxides. In particular, pristine graphene has been prepared in a rather elegant way by electrochemical exfoliation of graphite both in absence and in presence of surfactants to stabilize the suspension [23,24]. It is remarkable that in some specific media, using oxalic acid electrolytes, no surfactants are needed and suspensions of graphene are stable for years [24]. To this date, that significant suspended graphene has resulted in smaller amounts than traditional GO Hummers preparation [25] but may render interesting 3D forms in the future.

In addition to microstructure and charge capacities, materials used as electrodes have additional restrictions to retain biocompatibility during electric field application. It is crucial that the material hinders secondary radical formation reactions for example. Metals like Pt or stainless steel may be used as electrodes, but the electron-ion transfer at the surface induces H_2_O oxidation and O_2_ reduction, yielding to radical formation that results in enhanced inflammation and cell death. In vivo experiments show that the alive system protects itself from those effects by inflammation, encapsulating the implanted material, and eventually the implanted electrode needs to be removed.

An attractive alternative arises from the use of electroactive materials as electrodes, that allow redox intercalation within their structure, in a similar way to M^+^ ion batteries, since such redox processes offer an alternative to radical formation in aqueous electrolytes. That is indeed the mechanism working in IrO_x_ (really an oxohydroxide that allows intercalation of H^+^, Na^+^ and K^+^), polypyrrole or PEDOT polymers (allowing intercalation of cations and anions). Both material types have been studied as substrates for neural growth and as electrodes in electrostimulation [19,26]. Their charge capacity has been enhanced by specific preparation processes of dynamic electrodeposition [21,22] yielding one order of magnitude enhancement in charge capacity with respect to standard electrodeposited materials. However, it is possible in some cases to go beyond in charge capacity by nanostructuring the best materials in specific forms.

This work shows the most successful cases where such nanostructuring has led to enhanced charge capacities, and to significant electrostimulation effects, through the control of electrodeposition processes and modification of carbon nanostructures [18,24,27,28,29]. It also shows that such tuning does not always yields the best options, and that spontaneous chemistry may order the critical parameters that induce a specific nanostructure. Thus, while CNT and graphene generate IrO_x_-C hybrids with enhanced charge capacities, when PEDOT is added, as in trihybrid IrO_x_-CNT-PEDOT, the polymer encapsulates both IrO_x_ and CNT yielding final charge capacities related only to PEDOT [18]. On the other hand, if nitrogen-doped graphene oxide is used, the biocompatibility disappears, while the charge capacity has also been greatly enhanced [29].

Thus, optimal cases are found for the formation of hybrid nanostructured materials containing the best of both worlds, nanocarbons and IrO_x_. Charge capacities do not simply add, but the synergy among components increases their values to an additional order of magnitude, while compatibility is sustained under electric field application. Therefore, the stimulation times may be expanded significantly, and larger effects are reached in smaller times, while decreasing dramatically the side effects of electric field application.

The fundamental reason sustaining the peculiarity of these hybrids resides in the uniqueness of the properties of IrO_x_ in the nervous system mentioned above, and no other hybrids have been reported yet in those biological terms. When hybrids are formed with IrO_x_, a significant material in O_2_ reduction and water oxidation, or in biocompatible electrodes [21], charge capacities are found to increase several orders of magnitude in the nanostructured material beyond the values expected from the charge attributed to redox processes, thanks to the contribution of carbons. Those charge capacities remain stable during electrochemical cycling, due to the nanostructuring. The unique factor in the final materials comes from the fact that they maintain the redox intercalation properties and biocompatibility of the original material [18,24,27,28,29]. The underlying nanostructure is significant also from the fundamental chemical point of view. In the case of carbon nanotubes, the carbon tubes sustain the oxide as in a reinforced concrete structure [27], allowing thicker coatings and very stable electrodes to be used compared with pure IrO_x_. Graphene hybrids on the other hand offer a millfeuille structure that attains the same charge capacities. Nanocarbon hybrids containing layered graphene are also remarkably different to graphite hybrids in terms of reversibility. While graphene phases retain most of the capacity beyond 1000 cycles, in the case of larger carbon particles, the graphite hybrids, the charge capacity drops during the first 100 cycles to the original value for IrO_x_. The large size of graphite particles may favor carbon loss during cycling, probably through a smaller interaction with IrO_x_ [28].

Within that frame of thought, a further increase in charge capacities is also envisaged if nanocarbons include redox species that may contribute to the pseudo-capacity in the potential window available in aqueous solutions, without becoming hydrophobic. Indeed, some N-doped IrO_x_ hybrids enhance even further the charge capacity of the final material [29]. Beyond the enhancement of electrochemical delivery charge capacities, biocompatibility must be maintained in absence and presence of electric fields. Interestingly, the addition of N groups increasing even more charge capacities as mentioned above, leads to a chemical incompatibility in cell cultures.

Thus, as mentioned above, what follows summarizes the best biocompatible hybrid materials containing carbon nanotubes, and several graphene types, specifying how several components influence each of their properties. Within that range of phases, optimal new electrodes for electrostimulation are found. Those always involve IrO_x_ and nanocarbons, but specific groups or phases are not desirable, such as N doping of nanocarbons, while some components are detrimental in electrochemical terms.

## 2. IrO_x_ Basic Material: IrO_x_ Basic Electrodeposition Process

As mentioned, IrO_x_ anodically deposited is among the best conducting substrates for neural growth. IrO_x_, understood as a complex oxohydroxide of general formula K_1.7_IrO_0.8_ (OH) _2.2_ 1.8 H_2_O, had been anodically deposited before [20,21], and had distinguished from crystalline IrO_2_ or from anodic oxidation of metallic Ir metal. Anodic deposition of precursor solutions based on slowly hydrolyzed IrCl_3_ or IrCl_4_ solutions in alkaline conditions yields IrO_x_, yield coatings with poor adhesion and macroscopic cracks if constant current protocols are used [21]. However, dynamic pulsed anodic deposition renders thin layers (170 nm for 50 cycles and 300 nm for 100 cycles) of IrO_x_ that are well adhered and have a one order of magnitude larger charge capacity [21] than conventional IrO_x_ obtained by constant current deposition methods. The same dynamic deposition successful for IrO_x_ offers later additional mechanisms for the formation of hybrids. The final solid IrO_x_ coating is amorphous but contains K^+^ in a reproducible stoichiometry. Chemical exchange of K^+^ is possible and the ion is easily removed by soaking in water, and easily replaced by H^+^ or Na^+^.

**Figure 1:**
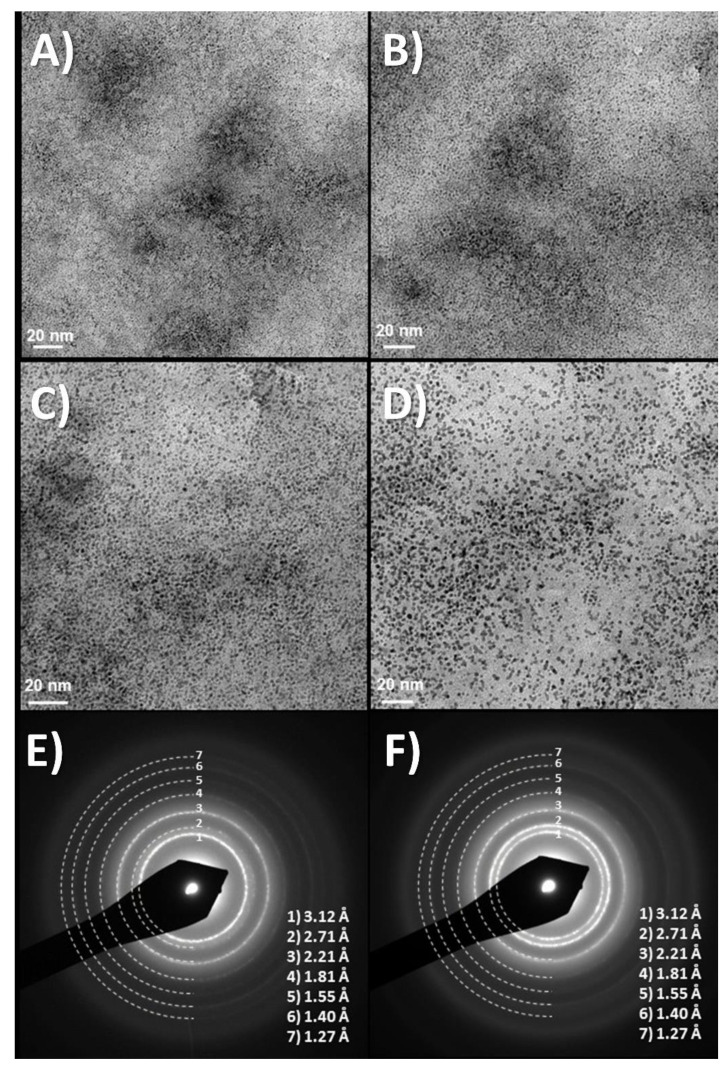
(**A**–**D**) Time evolution of TEM images at about 10 min intervals, showing TEM images of dry drops of Iridium oxo solutions obtained from hydrolysis of IrCl_3_, evolve under the electron microscope (120 KV Jeol). Last two images, (**E**,**F**) Diffraction rings obtained at this low resolution match those of quasiamorphous K_x_IrO_2_ and later metallic Ir. Thermal evolution has also been observed before in Ar atmosphere (in O_2_ yielding IrO_2_ rutile [21]) Images show two different time intervals. Global time in the order of minutes. (Original results).

**Figure 2:**
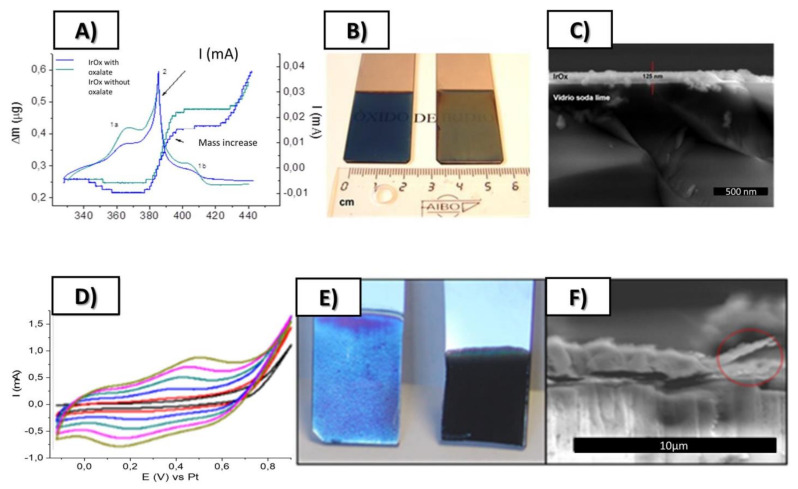
Top: (**A**) Simultaneous cyclic voltammetry and ECQM mass changes during dynamic Electrodeposition of IrO_x_ involving mass deposition and K^+^ intercalation/deintercalation, (**B**) SEM lateral images of resulting coatings on Pt (12 nm) glass slides, (**C**) Macroscopic images of IrO_x_ deposited on Pt (12 nm)-Ti (5 nm)-glass substrates. Bottom (**D**) Typical electrodeposition Cyclic voltammetry of IrO_x_-Nanocarbon hybrids (this case IrO_x_-NGO) (**E**) macroscopic images showing that the first layers are mostly IrO_x_ and (**F**) SEM lateral images of the coatings. From ref. [24,29] Published with permission of Elsevier.

It is worth remarking here that the nature of the Iridium precursor solution has been confusing in the literature [30], and the formation of hybrids discussed below benefits from such discussion. The existence of a nanoparticle suspension has been claimed upon aging of iridium solutions, based only on data from high-voltage TEM equipment. However, electron diffraction data from iridium solutions and coatings also show the existence of redox processes occurring under the electron beam. The existence of UV-VIS charge-transfer redox exchange conferring the blue color to the hydrolyzed solutions involve mixed-valence polynuclear Ir oxo species resulting from a hydrolysis process, and not necessarily nanoparticles of IrO_2_ [21]. Precipitation with excess K+ ions generates a solid with 2.2 K/Ir ratio, while anodic deposition yields a K/Ir ratio of 1.7 according to XPS [21]. That means that deposition is not a flocculation or electrophoretic process, but a true redox electrochemical process. Electrochemical quartz microbalance study also shows additional features for the deposition [21], where K^+^ is intercalated and deintercalated depending on the voltage applied during the process. Dynamic light scattering (DLS) measurements of the solution, yield similar cluster sizes found in initial low-intensity TEM measurements (10–20 nm) instead of the 2 nm size found in high-voltage TEM, which is truly a redox modification to metallic iridium (see Figure 1 and Figure 2). Careful interpretation of the global data allows us to identify that the electron beam acts as an electrochemical cell reducing Ir-oxo species containing K^+^ and OH^−^ to K_x_IrO_2_ and finally to Ir metal suspensions of 2 nm size nanoparticles, by a slow process for low-intensity electron beams, and very fast for high-intensity beams. Thus, the existence of iridium anionic oxoclusters in solution, formed during hydrolysis in a similar way to known polyoxometalates [31], is a more coherent explanation than thinking about generic nanoparticles. Therefore, as suggested above, the deposition of IrO_x_ is not an electrophoretic pure process of suspended IrO_x_ particles but, as other electrodeposition processes, is a full redox oxidation process of iridium oxoclusters in solution yielding an amorphous oxohydroxide.

That discussion is relevant in terms of possible nanocarbon IrO_x_ hybrid formations, since the nanostructure is dependent on the interactions among both components. It is also relevant because, although carbons do not deposit in absence of iridium, the deposition of IrO_x_ drives the deposition of carbons, by the existing interaction among them.

## 3. Comparison of Nanostructured IrO_x_-Nanocarbon Hybrid Materials for Various Nanocarbon Particles

Suspensions of carbon or nanocarbon particles, are remarkably stable in aqueous and organic solvents, and have been demonstrated to improve conductivity [1]. Recently the mechanism by which impedance decreases has been proven using macroscopic immersed pieces. Induced dipoles created in each conducting particle by an external applied field generate a physical contribution to charge transfer, and also favor chemical charge transfer mechanism that result in an enhanced effective area. Thus, carbon enhances conductivity not only when part of solid electrode materials, but also when in suspension in the electrochemical cells [32].

**Figure 3:**
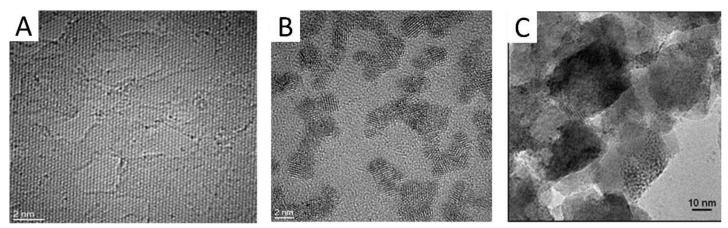
(**A**) Exfoliated graphite yielding pristine graphene and (**B**,**C**) adhesion of Ir oxoparticles adhered to electrochemically exfoliated graphene nanoparticles, eG ((**A**,**B**) HRTEM, (**C**) SEM). (Note that Iridium species evolve under the microscope).

When appropriate ions are present (ex. Mg^2+^), those nanocarbon suspensions may be deposited on electrodes through an electrophoretic/electrocoagulation mechanism [33]. Furthermore, when carbons are in iridium oxosolutions, a spontaneous adhesion of the oxo species to carbon seems to occur (ex Figure 3 showing iridium oxospecies adhered to pristine graphene) [24,27,28], possibly due to the dipolar interactions mentioned above. Carbons and nanocarbons defined in this work do not deposit from the ionic electrolyte in absence of iridium, but if iridium is already present, the deposition of IrO_x_ occurs with simultaneous deposition of carbon, in specific nanostructures. The driving process for the formation of the hybrid coating is, therefore, the deposition of IrO_x_.

It is also rather significant that when IrO_x_ is deposited in pure form the deposition is self-limited and maximum thickness around 300 nm are obtained, although the material is still conducting [21]. However, in presence of graphite or nanocarbons, there is not apparent limit for the deposition of the IrO_x_-C hybrid, even for the less conducting particles like graphene oxide.

Graphenes are truly a wide family of phases where structure and functionalization varies and microstructure is unique for each case. This work deals mostly with the most hydrophilic graphenes and nanotubes, usually functionalized with oxygen or nitrogen. However, additional options like pristine graphene are available. While graphene oxide (GO), highly defective and obtained by a strongly oxidizing Hummers method, is commercially available as powder in large amounts, the synthesis of pristine graphene is eluding such quantitative production. One of the most promising methods that may change that is the exfoliation based on electrochemical processes, that has reached significant results when graphite electrodes are used. Surfactants were used at the starting experiments [23], but exfoliation also renders stable graphene suspensions without surfactants [24]. In particular, oxalic acid solutions are remarkable since the resulting graphene suspensions are stable over years [24]. We have observed that oxalic acid oxidizes to CO_2_ during the application of the electric field, decreasing the electrolyte ionic strength and stabilizing the suspension of less hydrophilic pristine graphene.

In that particular case, the addition of iridium chloride to the exfoliated graphene suspension, and carbonate to raise the pH, still renders a stable suspension, that can be aged to allow slow iridium hydrolysis in the same way as pure iridium solutions. Figure 3B,C show HRTEM and SEM images of graphene particles (Figure 3A) to which iridium oxospecies attach (please note that iridium particles have reached the 2 nm scale mentioned above because of the electron beam).

Precipitation of the pure graphene suspension (eG), from exfoliated suspensions, may be achieved by addition of KI or KCl. The resulting solid contains labile oxygen attached to carbon according to XPS data. Such oxygen disappears when forming the hybrid with IrO_x_, which contains the same O/Ir ratio than pure IrO_x_, as shown in Table 1.

**Figure 4:**
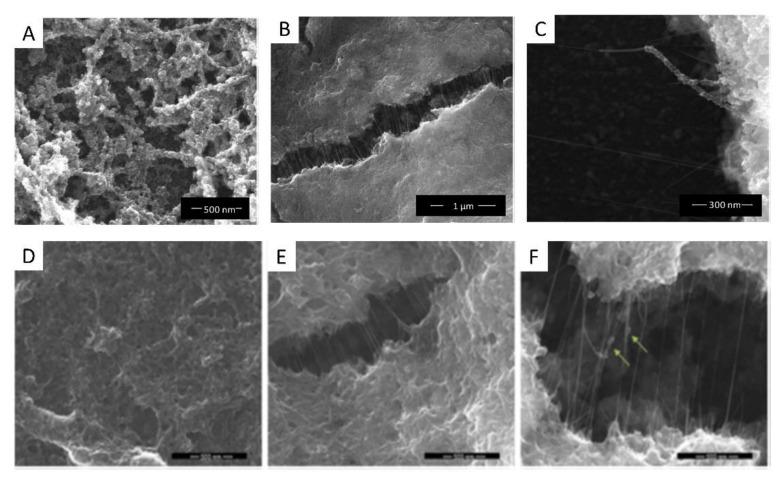
SEM images of IrO_x_-CNT (top **A**–**C**) and IrO_x_-CNT-PEDOT hybrids (bottom **D**–**F**) [18,27]. With permission from Elsevier.

**Figure 5:**
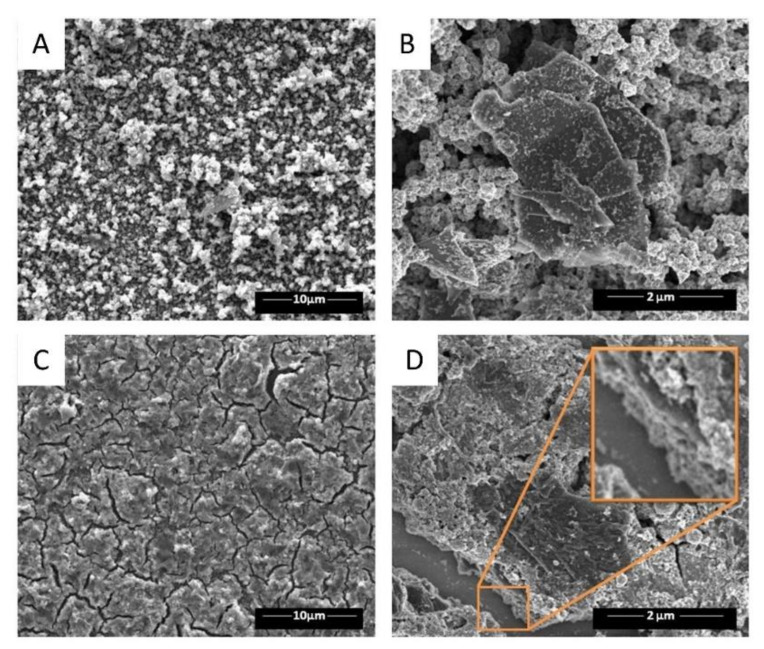
(**A**–**D**) SEM images for IrO_x_-GO hybrid at various scales showing typical cracks and the millfeuille nanostructure [28]. Yellow big square shows a magnified vision of the zone at small square. With permission from Elsevier.

**Figure 6:**
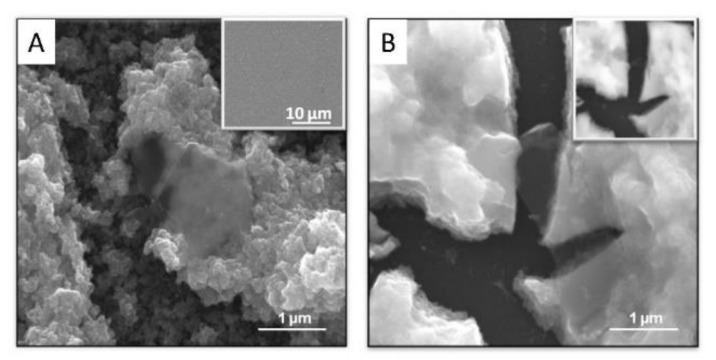
(**A**) SEM images of IrO_x_-eG hybrid showing the millfeuille nanostructure. (inset: macro scale showing the large homogeneity of the coating). (**B**) Crack developed under the SEM electron beam with millfeuille ordering. (Inset showing secondary electrons image, where carbon is not seen vs iridium) [24]. With permission from Elsevier.

**Figure 7:**
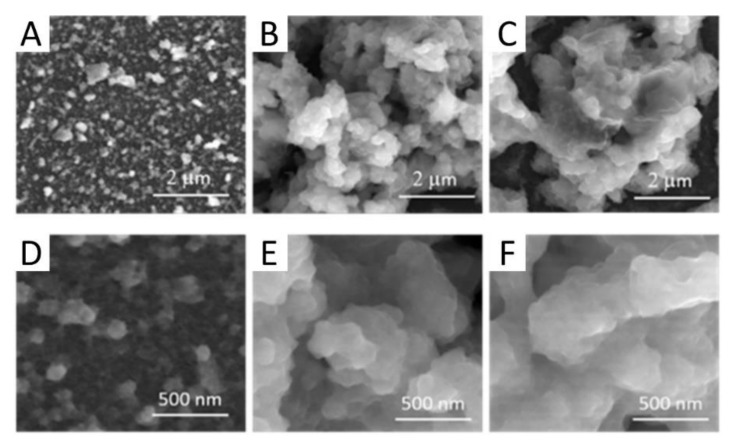
SEM images of IrO_x_-NGO hybrid coatings for one of the IrO_x_—NGO hybrid (NGO treated at 100 °C (**A**,**D**), 220 °C (**B**,**E**) and 300 °C (**C**,**F**) [29]. With permission from Elsevier.

As mentioned, the precursor solutions containing iridium oxospecies and nanocarbons get electrodeposited in all cases thanks to the driving force of IrO_x_ deposition. In general, the dynamic potential sweep must reach larger voltages (0.8 V for hybrids vs. 0.55 V vs. Ag/AgCl for IrO_x_). Figure 4, Figure 5, Figure 6 and Figure 7 show some representative images of the nanostructures observed for several of the hybrids prepared, containing CNT-COO-, GO, pristine graphene (eG) and N-doped GO (NGO). Graphite oxide may also be deposited as IrO_x_-graphite oxide, (not shown) but the final microstructure is more heterogeneous [27].

Composition and roughness for hybrids and pure components are summarized in Table 1 and Table 2. The roughness observed for the solid hybrid materials deposited on Pt (shown along other parameters in Table 2) depends on the carbon used, being in the order of microns for graphite oxide, graphene oxide and N-doped graphene oxide hybrids [24,27,28,29]. Large particles of graphite oxide are observed indeed in the IrO_x_-graphite oxide, while on the contrary, all IrO_x_-nanocarbons evidence a largely intertwined structure.

IrO_x_-CNT hybrid yields the clearer visualization of the interaction between iridium species and nanocarbons, since IrO_x_ is formed wrapping the nanotube. (Figure 4, and specially Figure 4C) [27]. When layered graphite or graphenes are used, the same adhesion is observed (see Figure 4) and an ordering resembling a millfeuille structure is found, with a stability that depends on the carbon particle size [24,28,29] (Figure 5, Figure 6 and Figure 7).

XPS surface analyses, even if taking into account the particular inner structure in IrO_x_-CNT hybrid, allow us to observe interesting features. Table 1 summarizes the stoichiometry ratios derived from quantification of those analyses reported in [24,27,28,29]. The K/Ir and O/Ir ratios evidence the existence of the same IrO_x_ in all of them, and only the hybrids containing GO or NGO, with a large oxygen amount show larger O/Ir ratios. The carbon content, above the endemic carbon found for pure IrO_x_, is larger for GO hybrids than for pristine graphene hybrids. The influence of this on the electrochemical properties is discussed below.

The nanostructure of the IrO_x_-CNT hybrid is retained if we introduce PEDOT in the hybrid, by simultaneous polymerization of EDOT during deposition. Thus, deposition in presence of EDOT results in a trihybrid, IrO_x_-CNT-PEDOT, very similar in nanostructure to the dihybrid (Figure 4D–F). Images show that the polymer seems to encapsulate CNT. As seen later, IrO_x_ is also encapsulated since the resulting voltammetries show only the behaviour of PEDOT-PSS and CSC_c_ values are of similar magnitude to those found for PEDOT-PSS, and therefore all gains found in IrO_x_-CNT are lost [18].

**Figure 8:**
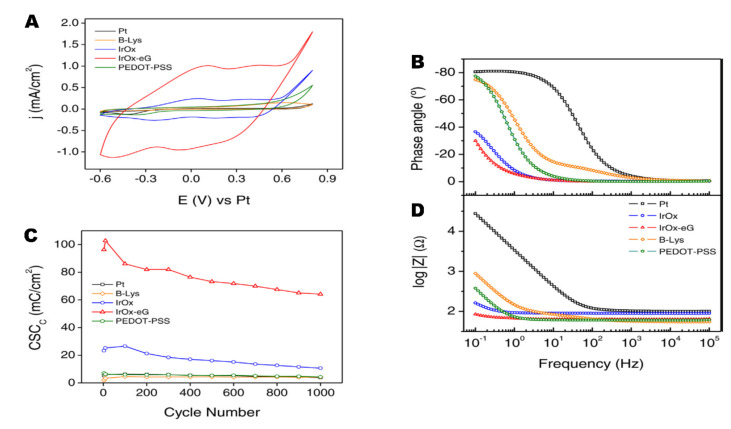
**(A**) Cyclic voltammetries in biologically emulating sodium phosphate buffer of representative nanostructured IrO_x_–graphene hybrids during electric field application in electrostimulation processes and (**B**) associated charge storage capacity changes during 1000 cycles. (**C**) Graphite hybrid, not shown sees the CSC decrease after a few cycles, to the IrO_x_ values. (**D**) Impedance comparison for several materials [34]. With permission from Elsevier.

Significantly, all two-component IrO_x_-nanocarbons hybrids show the quasireversible reduction and oxidation waves described for IrO_x_ in cyclic voltammograms [21,24,27,28,29], involving redox intercalation and deintercalation of M^+^ and OH^−^ from the amorphous structure. (See Figure 8). Additionally, the initial electrochemical properties of all those hybrid coatings render similar enhancement of charge capacity values, (see Table 2 and Figure 8) with respect to IrO_x_, independently of the nanocarbon used. Even for graphite, when thickness is similar, values are near 100 to 130 mC/cm^2^ as for nanocarbons containing oxygen or pristine graphene [24,27,28] (vs. 170 mC/cm^2^ if they contain nitrogen and oxygen [29]). However, for the IrO_x_-graphite case, such charge capacity is lost almost immediately with cycling voltammetries and returns to the 10–20 mC/cm^2^ values observed for pure IrO_x_. Graphene hybrids retain 70% of the capacity after 1000 cycles, while CNT hybrids see their CSC values decrease slowly.

If we include PEDOT, and form the trihybrid IrO_x_-CNT-PEDOT, the voltammetries do not show anymore the IrO_x_ waves, and the CSC values drop to those found for PEDOT, as shown in Table 2. Raman data from this thermally treated hybrid, however, show that the three components exist [18], and that such low capacity is not due to absence of any component. Thus, the electrochemical behavior confirms the encapsulation suggested by the SEM images. Furthermore, in terms of biocompatibility, the effect of PEDOT also changes the outcome of primary neuron cell culture, although it remains biocompatible when astrocytes or cocultures of both are used [18], which is a behavior similar to the one found for the polymer. All evidence suggests, therefore, encapsulation by the polymer.

**Figure 9:**
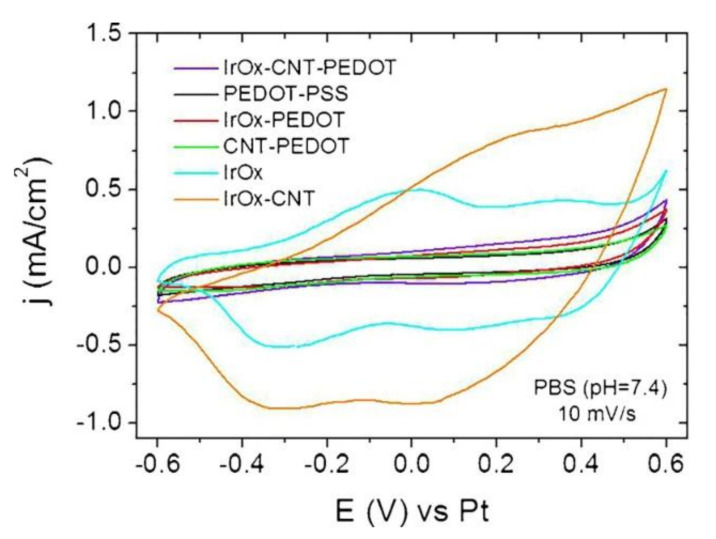
CV showing the significant decrease in current when PEDOT is incorporated to the IrO_x_-CNT hybrid. (IrO_x_-CNT compared with IrO_x_-CNT-PEDOT and with individual components). The lowest currents always correspond to the composites or hybrids containing PEDOT [18]. With permission from Elsevier.

It is also remarkable that the impedance of IrO_x_-graphene hybrids is very low at low frequencies [34]. (See Figure 8 and Figure 9). For both DC and AC electrostimulation in the nervous system, this factor is essential, since it would involve less heating, and a faster response of the electrodes.

Nanostructuring hybrids based on IrO_x_ with nanocarbons offers clear advantages with respect to macroscopic graphite hybrids, preserving and enhancing the electrochemical properties of IrO_x_. In the case of N-doped GO hybrids, an enhanced capacity seems to be derived from the fact that nitrogen also sees its oxidation state modified during cycling. In some cases, for N-doped GO hybrids prepared at low temperature, with N groups considered reduced (amines, for ex), CSC increases because of such redox changes, and seem to achieve CSC values corresponding to more oxidized hybrids. Significantly enough, Nitrogen reaches even the nitro state, and yields the highest CSC measured in these hybrids and has a large stability. It seems reasonable to assume that such nitro stability is related to the existence of the Ir environment.

If we consider the carbon content for each of the hybrids, as obtained from XPS analyses [21,24,27,28,29], it is evident that not all nanocarbons contribute the same amount to the final charge capacity (Table 1 and Table 2). IrO_x_-GO has values about 1.7 C/Ir, while IrO_x_-eG has 0.4 C/Ir, and both reach very similar CSC. While these proportions are related to the amount of graphene in suspension during preparation, which is lower for exfoliated eG, it is significant. Pristine graphene confers four times larger effect per carbon atom to the CSC of the final hybrid than GO.

A side question arises here: since large size distribution of particles of GO, N-GO, CNT-COOH, involve less reproducibility of the actual carbon particles that get deposited. Control is not easy and strong stirring is needed in those cases. Correspondingly, the final parameters found for them have a larger spread. eG suspensions, on the other hand, have a narrower size distribution [24], and the final hybrid has lower roughness and a more reproducible nanocarbon content.

In general, cycling is also similar among hybrids. All hybrids retain 70 to 80% of the original capacity after being cycled in the aqueous potential window a thousand times. Only N-doped graphenes have different behavior, even among them, because N groups oxidize in different extents during the cycling process. In the case of the larger oxidation, the NGO treated at 300 °C prior to the formation of the hybrid, the hybrid resembles the other graphene oxides hybrids. In that particular case, the anodic deposition process seems to generate a nitro functionalization in the nanocarbon and in the hybrid, as mentioned above, which is rather unusual, but it results stable over cycling.

## 4. On Biocompatibility of IrO_x_-Nanocarbon Hybrids as Substrates for Neural Growth

**Figure 10:**
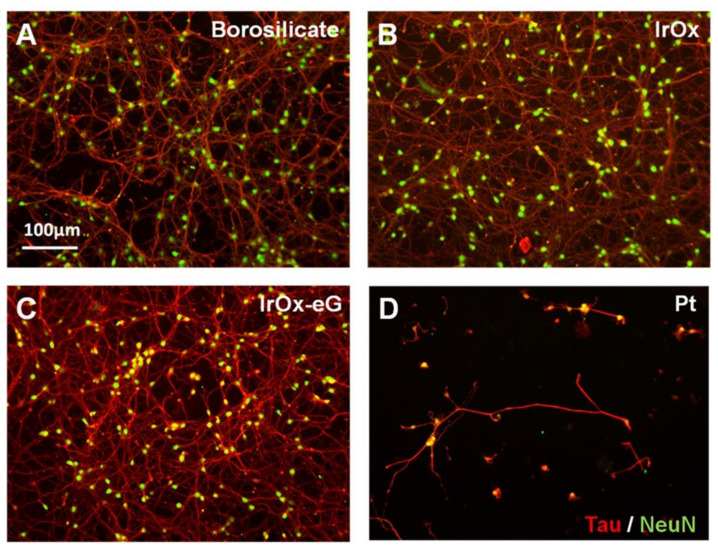
Neuronal survival and functionality on IrO_x_-eG. Representative fluorescent microphotographs of neurons growing on: (**A**) Borosilicate glass, (**B**) IrO_x_, (**C**) IrOx-eG and (**D**) Platinum, stained against Tau (red) and NeuN (green) showing dendrites and living cell nucleii. Scale bar = 100 µm [24]. With permission from Elsevier.

**Figure 11:**
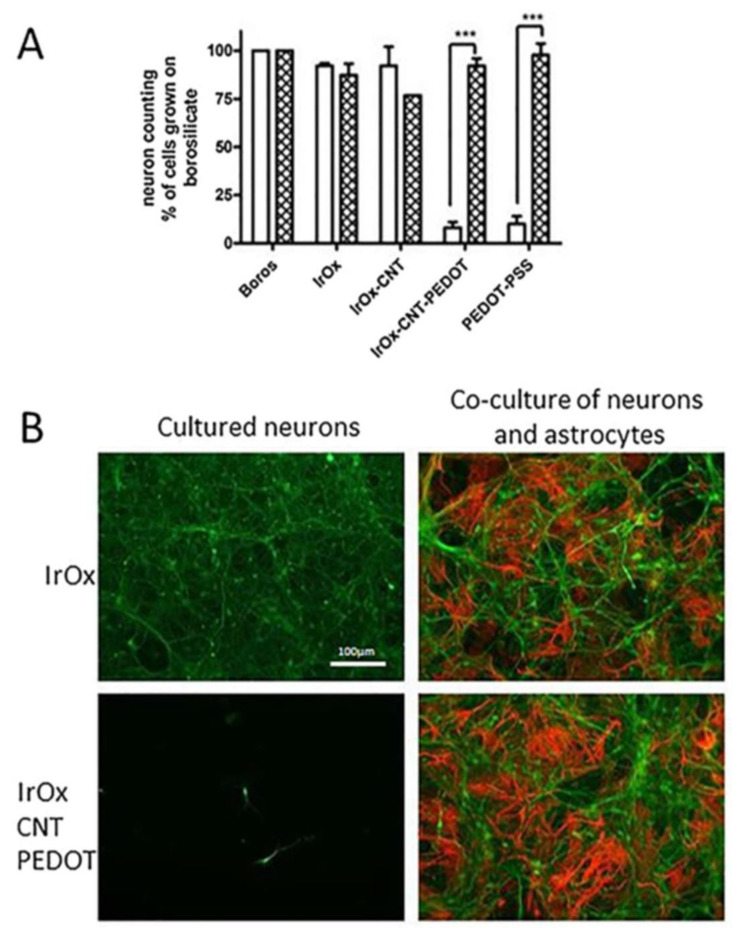
Astrocyte influence on neuronal survival on IrO_x_.nanocarbon nanostructured materials containing conducting polymer PEDOT with respect to IrO_x_. (**A**) Dissociated neural cells were (seeded at 115,000 cells × cm^−2^ on different materials coated with poly-L-lysine (white bars) or with a monolayer of confluent astrocytes (cross bars) and grown for 5 DIV. (**B**) Representative fluorescent photomicrographs of primary cultures of enriched neurons and of neuron-astrocyte co-culture growing on IrO_x_ and IrO_x_-CNTPEDOT. Cells were processed for tau (green) and GFAP (red) immunocytochemistry to label neurons and astrocytes, respectively. Scale bar = 100 μm [18]. *** *p* < 0.001 vs. neurons grown on poly-L-lysine coated materials. With permission from Elsevier.

Figure 10 and Figure 11 show neural cell cultures on some representative hybrid IrO_x_-nanocarbon materials on primary mouse neuron cell cultures and cocultures of neurons and astrocytes. It results obvious that primary cells grow very well in IrO_x_ and IrO_x_-nanocarbons hybrids, while they do not in PEDOT-PSS containing hybrids. However, when astrocytes are part of the cell culture, the polymer does not have any influence on primary cell survival. Additional data show that inflammation is even lower in cocultures if the polymer is present. The complexity of the model adequate for neural growth is evident. The most restrictive cell cultures based only on primary neurons differentiate more among materials than the coculture of neurons and astrocytes, and evidence that viability of neurons is easier in IrO_x_ and IrO_x_–nanocarbons hybrids than in any other substrate [18,21,24,27,28,29]. Other reports show also viability in graphenes [11,17].

Comparison of neural cell culture in IrO_x_ and several types of hybrids suggest that those containing pristine graphene, CNT or GO retain the optimal behavior as substrates [24,27,28]. It also shows that all IrO_x_-NGO hybrids do not allow cell growth. PEDOT containing hybrid, as pure PEDOT-PSS, does not allow cell growth if only primary neurons are used, but is a good substrate in more realistic astrocytes-neurons culture [18] or in xenopus cell growth [13]. Cell cultures, therefore, also show that the trihybrid nanostructured material including EDOT yield a phase where the only exposed surface is the polymer, evidencing encapsulation of other components by the polymer. CSC enhancement observed for the IrO_x_-nanocarbons disappears in such case and only PEDOT-PSS electrochemical contribution appears. Therefore, it is not only nanostructure what confers specific properties to the hybrid, but the basic chemistry of the components that is retained upon formation of the hybrid. The hierarchical order, yielding encapsulation in the case of PEDOT, is crucial. Despite the enormous popularity of PEDOT-PSS, its contribution is detrimental to the final effects on primary neurons, and on electrochemical features. However, it is also known that counterion may modify the effect. Bilayers of Polypyrrole on PEDOT where X is an amino acid molecule (or other biological active species) are better than either the polypyrrole or PEDOT components, evidencing the need for additional work in those systems [22]. On the other hand, IrO_x_-nanocarbon hybrids are to date the best active electrodes or electrode coatings in terms of repair. IrO_x_-CNT, IrO_x_-GO and IrO_x_-eG are good as substrates for mammalian neural cell growth even in the stricter neural cell cultures.

## 5. On Electrostimulation and Neural Repair

**Figure 12:**
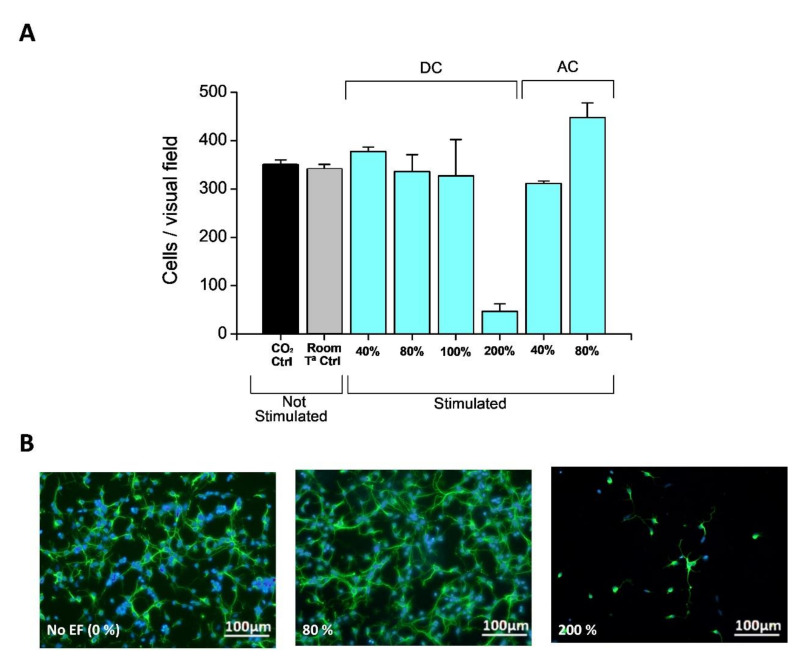
(**A**) Quantification of neural cell growth in absence and presence of EF with Q delivered below (80% of the total CSC) and above CSC (based on Tau imunostaining of scratched cortical neuron cultures showing spontaneous regeneration of surface covered by new neurites after scratching the neuronal monolayer at 5 DIV in each case) [34]. (**B**) Representative images of cell growth with no field and using (80% and 200% of the CSC value for the charge delivery). (**B**) Neurite and nuclei images for comparison (Cells were processed for Tau inmunocitochemistry and Bis-benzimide staining of the nuclei). With permission from Elsevier.

Once an electric field is applied, even for optimal substrates, the final viability of cells depends on the amount of charge delivered (See Figure 12) [34]. When the charge delivered is above the charge capacity CSC threshold, neural cells survival drops to 20% of the original population. Actual field application and reporting the total charge delivered is not a usual report in bioelectrodes, and no easy comparison can be established with other works at present, but the check on the threshold CSC limitation suggests that the main factor contribution from these electroactive materials is the redox intercalation, as proposed in the introduction.

**Figure 13:**
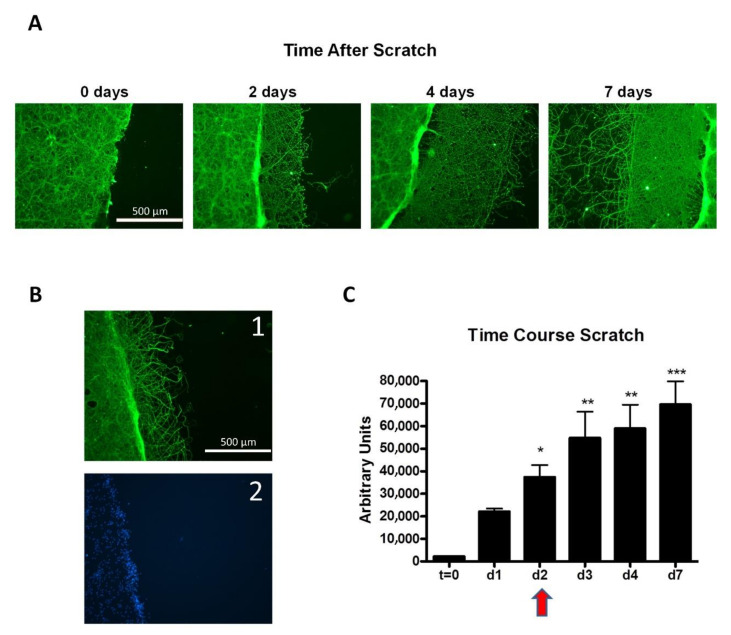
Spontaneous regeneration of an in vitro “wound like” scratch in neural cell cultures over IrO_x_-eG hybrid. (**A**) Tau imunostaining of scratched cortical neuron cultures showing spontaneous regeneration of surface covered by new neurites after scratching the neuronal monolayer at 5 DIV. (**B1**) Neurite and (**B2**) nuclei images for comparison (Cells were processed for Tau inmunocitochemistry and Bis-benzimide staining of the nuclei). (**C**) Quantitative integration of the area covered by new neurites at different days after scratch. Results are mean ± sem (n = 5). * *p* < 0.05; ** *p* < 0.01, *** *p* < 0.001 vs. t = 0 after significant one-way ANOVA. Electric field application would be carried out in a zone where a more drastic change is seen for growth (d2, marked in the red arrow) [34].

Furthermore, additional models for repair may be clarifying the role of electrode materials. Focusing only on neural repair, a physically created scratch in a stable neuron cell culture is a very representative model for injury and wound repair, in absence and in presence of applied electric fields. Figure 13 shows the spontaneous repair (visual and quantified) of an induced scratch emulating a “wound like” cell culture, over IrO_x_-eG hybrid. It can be observed that within days, dendrites fill the void space, being the biggest change around day 2.

**Figure 14:**
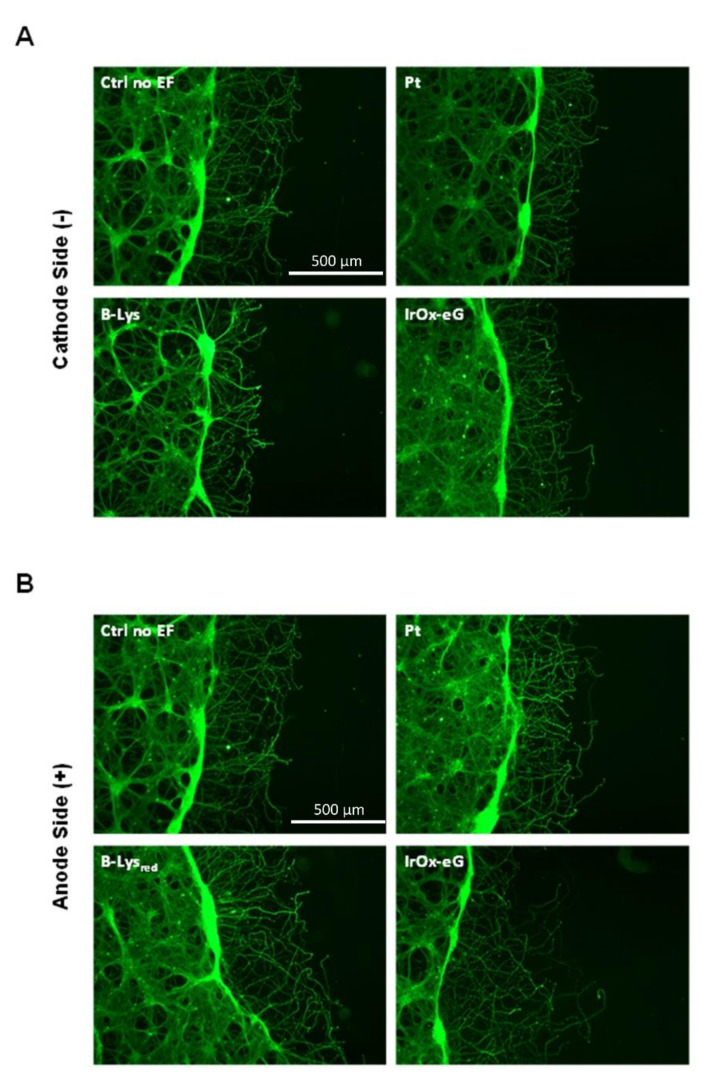
Scratch model repair under the application of an electric field using IrO_x_-graphene as substrate, (**A**) cin cathode and (**B**) anode areas. The firs image in each set corresponds to the reference spontaneous scratch growth using polypyrrole surface on PEDOT with lysine counterions. (Note that cells are near polypyrrole and not PEDOT in the polymer bilayer). Scale bars are equal for all images. [22] B-lys is a PEDOT-Polypyrrole bilayer with lysine counterion, IrO_x_-eG is the hybrid described in this work. With permission from Elsevier.

When an electric field is applied, however (see Figure 14), the results depend on the substrate electrode used. Recent reports show that repair (measured as dendrite filling of the void space created), is decreased by using electrostimulations with Pt electrodes. However, they remain nearly equal to the spontaneous neuron growth for biocompatible polymers that have CSC values near that of IrO_x_ [34]. When the IrO_x_-graphene hybrids, with one order of magnitude larger CSC are used, however, even for the same charge delivered, a larger repair is observed in both the cathode and the anode side. Furthermore, since CSC values are so much larger, longer electrostimulation times are also possible, suggesting a longer available electrostimulation time for implanted electrodes based on these materials.

**Figure 15:**
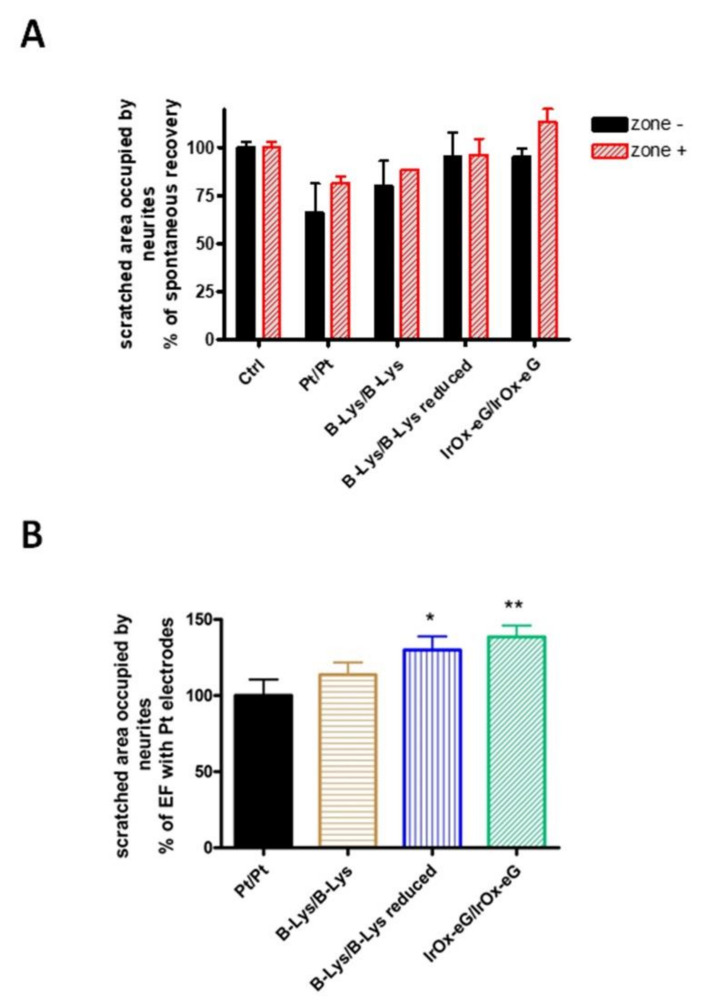
(**A**) Quantification of scratch repair (as area reoccupied by neurites) for various pairs of electrode materials, using symmetrical cells (equal electrodes), or reducing first the electrode that will act as anode, as compared with spontaneous repair [34]. Results are mean ± sem. * *p* < 0.05 and ** *p* < 0.01 after significant one-way ANOVA (*p* < 0.05; F3,15 = 3.486) and post-comparison tests. (**B**) Quantification of scratch repair (as area occupied), as compared with the use of Pt electrodes. With permission from Elsevier.

Concerning the electrochemical cell configuration used to achieve such electric field application, there are several ways to enhance the global cell charge capacity during electrostimulation. Using asymmetric electrochemical cells where the anode has been previously reduced allows for an additional global charge delivery of the cell. In such a case (see Figure 15), the final observed repair is also larger, even for the same charge delivered.

Furthermore, comparing neural repair in the anode and cathode sides in any of the possible combinations of electrodes reported [34], the anode systematically shows a better result for neurite filling of the scratch (see Figure 14). Such differences suggest that the electrochemical reductions possible at the cathode may be detrimental. In particular, iridium may be reduced and ions from the electrolyte be consumed since they intercalate within IrO_x_, although the calculated depletion of extracellular Na^+^ ions is very small. Additionally, and more relevant, is the possible side reaction involving dissolved O_2_ reduction, that may generate oxo radicals. No evidence exists yet to elucidate which of those mechanisms is the significant one, although is clear that the effect is much lower for these hybrids than for Pt electrodes.

In either case, as quantified in Figure 15 for a number of coupled anode/cathode systems, repair in presence of electric fields is well beyond the spontaneous one for the IrO_x_-eG hybrid electrodes, while in the case of Pt electrodes lower repair below the spontaneous one. The repair in the case of the hybrid is also above the lower CSC polypyrrole-PEDOT-lysine results, resulting in a significant outcome for the new electrodes [34].

## 6. Conclusions

Electrostimulation electrodes find a new family of nanostructured coatings, IrO_x_-nanocarbons, containing nanotubes, graphene or graphene oxide, that allow much larger charge delivery, without damaging effects, and with proven regeneration effects in very short times in the nervous system.

From a fundamental point of view, it is remarkable that the IrO_x_ environment allows a significant wide span of oxidation states for nanocarbons, from labile oxygen in exfoliated graphene to nitro graphene, and with that a wide range of redox properties that contribute to the large CSC values found. Significantly enough, when considered per carbon atom, exfoliated pristine graphene is the nanocarbon that contributes more to the charge capacity and also the one that allows a larger reproducibility and impact in regeneration.

Additional components that may favor softer and mechanically appropriate electrodes, like conducting polymers, decrease the compatibility for primary neurons, although are optimal for a more realistic model of astrocytes-neurons cocultures. In particular, the polymer forms around other components, yielding to their encapsulation, and isolation from the electrolyte media, and decreasing the charge capacities to those of the polymer alone. N-doped graphenes, on the other hand, may reach the nitro-graphene state when part of the IrO_x_-graphene hybrid, and while increasing their CSC even more, result in non-biocompatible materials.

Thus, IrO_x_-nanocarbon hybrids are unique at present, in modulating the redox range available, but also in the nanostructure found, which is governed mostly by the nanocarbon structure, and the interaction with the iridium oxohydroxide. A reinforced concrete -type nanostructure or a millfeuille nanostructure are found, that are stable over more than a thousand cycles, and with similar charge capacities for acting within the range of potentials are for aqueous and biologic solutions.

From a more applied point of view, the resulting impedance of coatings derived from them are significantly lower and favor electrostimulation minimizing greatly the detrimental effects found for pure metals. In all cases, such conductivity also allows for bipolar electrochemical use, without electrical contact, as already proven for IrO_x_ [13], and work continues being developed in their use as electrostimulation electrodes with and without contact.

In conclusion, microstructural and chemical factors along with dipolar interactions determine the nanostructure achieved and the final electrochemical properties that have allowed the creation of new biocompatible materials based on nanocarbons and IrO_x_. In turn a new range of charge delivered in a biological system is possible. Regeneration is being observed in short times, and with very long times of stability of the electrodes. That, in turn, offers new clinical applications in electrostimulation, beyond the individual graphene or IrO_x_ components.

## Figures and Tables

**Table 1:** Atomic ratios based on XPS analysis for IrO_x_ and IrO_x_-nanocarbon hybrids. Data from ref. [21,24,27,28,29].

Material	C/Ir	O/Ir	K/Ir	N/Ir	N/C	O/C
IrO_x_ [21]	1.2	3.6	1.8	-	-	3.0
IrO_x_-CNTO [27]	1.4	3.9	1.8			3.5
IrO_x_-GO [28]	2.9	5.1	1.7	-	-	1.7
IrO_x_-eG [24]	1.6	3.5	1.8	-	-	2.2
IrO_x_-NGO300 [29]	4.0	9.2	1.7	0.7	0.18	2.3

Note that data from IrO_x_-CNTO has the larger error due to the intrinsic inner structure holding the CNT, and IrO_x_-CNT-PEDOT structure prevents these analyses. (- symbol meaning no data may exist for that, since the material does not contain that element).

**Table 2:** Thickness, roughness and charge capacities for each hybrid, vs. pure IrO_x_ and other materials.

Sample	Thickn. (µm)	RMS (µm)	C_grap_/Ir	CSC_c_ (mC/cm^2^)	CSC_c_ (mC/cm^2^)/C_graph_	CSC_c_ F/g	CSC_c_(F/g) /C_graph_
IrO_x_ [8]	0.14	0.002	0	22	22	392.9	-
IrO_x_-CNT	1.4	0.55	-	80–100	-	-	-
GOIr [10]	1.5	2.4	1.7	108	64	257.1	151
eGIr [11]	0.7	0.3	0.4	94	235	479.6	1200
IrO_x_-NGO 300	4.0	4.1	0.7	177	63	158	56
IrO_x_-CNT- -PEDOT PSS	2–3	0.5-1	-	23	-	-	-
PEDOT-PSS	1–3		-	13	-	-	-

(- symbol meaning no data may exist for that, since the material does not contain that element).
